# Panuveitis as a manifestation of ocular syphilis leading to HIV diagnosis

**DOI:** 10.4103/0974-620X.60019

**Published:** 2010

**Authors:** Jay Kumar Chhablani, J. Biswas, S. Sudharshan

**Affiliations:** LV Prasad Eye Institute, Hyderabad, India; 1 Sankara Nethralaya, Chennai, India

**Keywords:** HIV, ocular syphilis in HIV, syphilitic panuveitis in HIV

## Abstract

Syphilis is a rare cause of panuveitis. We present the case of a 33-year-old man who presented with diminution of vision of three months duration in his left eye (OS), without any other systemic illness. Ophthalmic examination showed features of pauveitis with dense vitreous exudates, disc pallor and sheathing of vessels on fundoscopy. A diagnosis of probable endogenous endophthalmitis was made and vitreous tap performed. Vitreous biopsy showed no growth of fungus or bacteria. Rapid plasma reagin (RPR) and Treponema pallidum hemagglutination (TPHA) test were positive. Enzyme-Linked Immuno Sorbent Assay (ELISA) and Western Blot test were then performed, which revealed concurrent HIV infection. The patient improved dramatically with intravenous penicillin therapy. HIV positive patients may present with panuveitis secondary to ocular syphilis, as the only presenting feature in HIV positive patient in absence of any other systemic features.

## Introduction

Manifestations of ocular syphilis in HIV-infected patients include iridocyclitis, papillitis, optic perineuritis, retrobulbar opic neuritis, branch retinal vein occlusion, chorioretinitis, periphlebitis, serous retinal detachment.[1] Syphilis is an uncommon cause of uveitis in HIV infected patients.[2] We report a patient primarily diagnosed as syphilitic uveitis, who on further investigations was found to be HIV positive. To our knowledge, there are no previous reports where syphilitic panuveitis led to diagnosis of HIV infection.

## Case Report

A 33-year-old male presented with complaints of diminution of vision and floaters in the left eye (OS) for last three months with no other systemic complaints. The patient denied history of multiple sex partners and blood transfusion.

**Figure 1:**
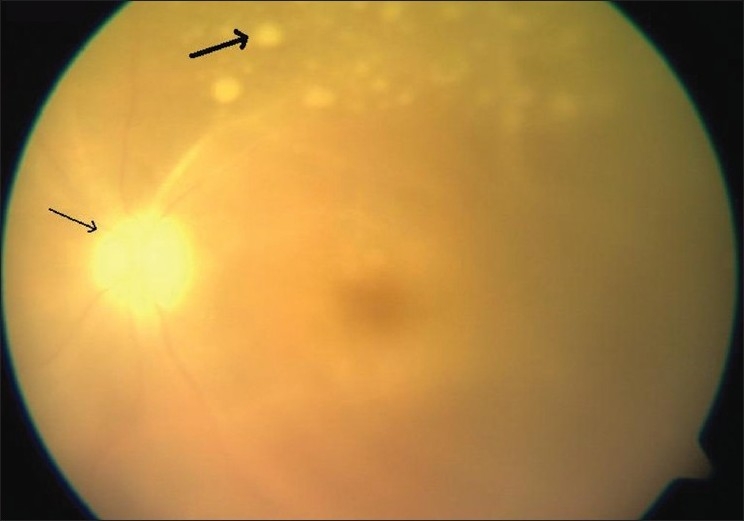
Fundus picture at presentation shows dense vitreous exudates (thick arrow) with disc pallor (thin arrow) and sclerosed vessel

On examination his best corrected visual acuity was 6/6; N6 in the right eye (OD) and hand movements in (OS). Ocular examination was unremarkable OD. Examination OS showed fine pigmented keratic precipitates, aqueous cells and flare grade 1, sluggishly reacting pupil with pigments on the lens. Intraocular pressure (IOP) with applanation tonometry was 10 mmHg both eyes (OU). Fundus examination OS showed hazy view due to dense vitreous exudates with disc pallor and sclerosed vessel [Figure 1]. Systemic examination was unremarkable. Hematological investigations showed Erythrocyte Sedimentation Rate (ESR) of 30 mm first hour, negative Polymerase Chain Reaction (PCR) for toxoplasma, herpes simplex virus, herpes zoster virus, and cytomegalovirus. Serum Angiotensin Converting Enzyme (ACE) and Cerebrospinal fluid (CSF) parameters were within normal limits. HLA- B5 was not detected.

A clinical diagnosis of panuveitis (anterior uveitis, vitritis, retinal vasculitis) secondary to endogenous endophthalmitis or sarcoidosis or Behcet′s disease was done. Vitreous tap was done, which showed no growth of any bacteria or any fungus. At this stage extensive laboratory investigations were ordered. RPR (1:32) and TPHA (1:640) were positive. ELISA and Western Blot test for HIV were also positive, with a CD4 count of 187 cells/μl (normal 383-1347 cells/μl). On repeated questioning, the patient gave a history of multiple sexual partners. He was administered intravenous penicillin G 24 million units/day for 14 days, and intramuscular benzathine penicillin 2.4 million units weekly for three weeks. He was also started on Highly Active Antiretroviral Treatment (HAART) by the infectious disease specialist.

**Figure 2:**
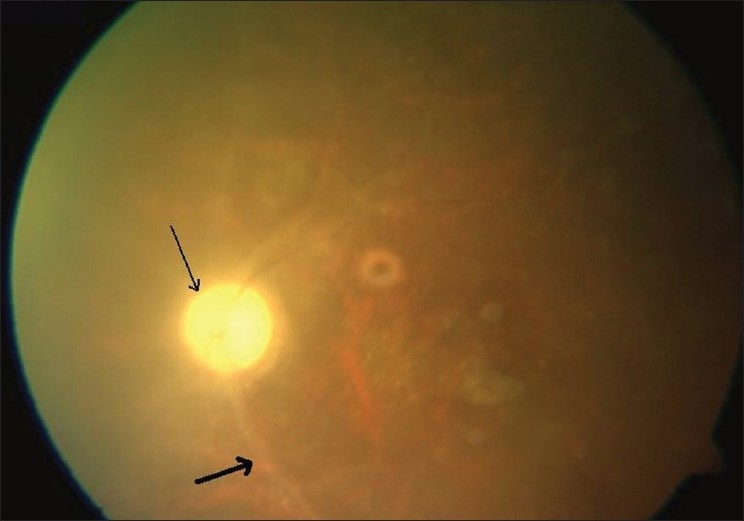
Fundus picture at last visit shows sclerosed vessels (thick arrow) and disc pallor (thin arrow)

At the one month follow-up visit, visual acuity OS improved to 6/60; N18. Examination OS revealed a quiet eye with grade one vitreous haze. At his last follow-up visit at two years (24 months) after onset of symptoms), visual acuity OS was 6/60; N36. Anterior segment examination was unremarkable except sluggishly reacting pupil and early posterior subcapsular cataract. Fundus examination showed disc pallor, sclerosed vessels with attached retina [Figure 2]. The patient was advised regular follow-up and continued antiretroviral treatment.

## Discussion

Ocular syphilis is a rare manifestation of syphilis (6-9%)[3] and was reported to have a prevalence rate of 0.6% in HIV-infected patients.[4] The increasing incidence (9%) in post-HAART era indicates that the immunological reconstitution after HAART is not protective.[5] Ocular syphilis is more rapidly progressive and more extensive in HIV infected patients in comparison to HIV uninfected patients.[6] It is usually bilateral and more common in males.[4]

Panuveitis as the only presentation of ocular syphilis, leading to diagnosis of HIV, is rare.[1,2] As syphilis is the most common bacterial eye infection in HIV-positive patients, all HIV- positive patients with uveitis should be tested for syphilis and vice versa.[4] Specific treponemal serum antibody tests such as FTA (Fluorescent Treponemal Antibody) - ABS (Absorption) are sensitive and specific but do not provide information on disease activity. Nonspecific serum antibody tests such as RPR and Venereal Disease Research Laboratory test (VDRL), however, are more useful to obtain information on disease activity and therefore for therapeutic monitoring. Up to 38% of HIV positive individuals can be seronegative for specific treponemal serological tests despite active syphilitic disease.[2] It is recommended to check serum nontreponemal titers at intervals of 1 month, 2, 3, 5, 9 and 12 months to ensure that titers are declining appropriately (at least four fold in three to five months).[1]

Ocular syphilis is not correlated with CD 4 counts.[3–5] Usually CD4 counts are in low normal range.[1,4] Kooed *et al*. showed that primary and secondary syphilis was associated with a decrease in CD4 cell counts and increase in HIV-RNA level. These levels returned to presyphilis levels or improved after treatment for syphilis.[7]

Central nervous system involvement is more frequent in HIV-positive patients[6] and is associated with CSF abnormalities (pleocytosis,[4] elevated proteins)[1] and neurological manifestations.[4] On the contrary, some reports have showed absent pleocytosis and negative VDRL on CSF examination.[8] Although the CSF analysis in patients with ocular syphilis does not change the treatment regimen, but helps to quantify the activity of the disease.[5] Four indications for lumbar punture have been proposed: (1) syphilis with neurologic abnormalities, (2) before retreatment of a patient with a relapse,(3) as a baseline before treatment with a nonpenicillin regimen, (4) in infants suspected of having congenital syphilis.[8] We perform CSF analysis in all patients with syphilitic uveitis, retinitis, or optic neuritis. Standard treatment of ocular syphilis is intravenous penicillin, as recommended for patients with neurosyphilis, regardless of immune status.[2] Relapse following intramuscular penicillin therapy is not rare.[2] Although the recommended penicillin (or ceftriaxone) intravenous therapy is usually effective in restoring complete visual function, reinfection by treponema pallidum may occur and a relapse of ocular manifestations is obscured in up to 14% patients.[8] Available alternatives, for use in penicillin allergic patients, include doxycycline, chloramphenical and ceftraixone because of their erratic CNS and ocular penetration, erythromycin and tetracycline should be avoided.

Fathilah *et al*. report a case of Jarisch-Herxheimer reaction in ocular syphilis.[9] Usually this reaction is self limiting but use of intravenous methylpredinoslone[10] or low dose steroids[11] have been reported. Syphilitic panuveitis may be the only presenting feature in HIV patients. Early diagnosis and therapy with intravenous penicillin and antiretroviral therapy will result in substantial visual recovery. Combined management with genitourinary physician for the systemic disease, including HIV, is recommended. Long term ocular and systemic follow up is mandatory because relapse or reinfection may occur.
